# Retiring for the night: how negative attitudes towards ageing can shape expectations and attitudes towards sleep among adults aged 60+

**DOI:** 10.3389/fpsyg.2025.1556248

**Published:** 2025-04-30

**Authors:** Serena Salvi, Jennifer Murray, Dilek Önkal, Valerie Egdell

**Affiliations:** ^1^International Observatory on End of Life Care, Lancaster University, Lancaster, United Kingdom; ^2^School of Health and Social Care, Edinburgh Napier University, Edinburgh, United Kingdom; ^3^Newcastle Business School, Northumbria University, Newcastle, United Kingdom

**Keywords:** ageism, sleep quality, dysfunctional beliefs towards sleep, sleep, older adults

## Abstract

**Introduction:**

Negative attitudes towards ageing are pervasive and can create significant barriers to quality of life and longevity for adults aged 60 and older. Studies indicate that older adults with negative views of ageing often perceive common, treatable ailments as a natural consequence of age, impacting their health-seeking behaviours, such as good sleep hygiene. Little is known about how these ageing attitudes shape beliefs about sleep in older adults.

**Methods:**

This study examined the association between attitudes towards ageing and beliefs about sleep in older adults, using data collected through online surveys. Measures included the Pittsburgh Sleep Quality Index, the Centre for Epidemiologic Studies Depression Scale, and the Brief Ageing Perceptions Questionnaire. Hierarchical regression analysis was applied to assess the predictive relationships among these variables.

**Results:**

Emotional responses to ageing, such as worry and frustration, were strongly associated with dysfunctional sleep beliefs, explaining additional variance in sleep beliefs even after controlling for demographic factors, depressive symptoms, and sleep quality. Control-related perceptions of ageing predicted locus of control for sleep, with positive beliefs associated with an internal locus of control and negative beliefs linked to an external locus of control.

**Conclusion:**

Findings suggest that perceptions of ageing significantly influence attitudes towards sleep, potentially affecting health-seeking behaviours. Addressing age-related stereotypes and fostering positive attitudes may reduce barriers to healthcare engagement and encourage healthier sleep behaviours in older adults, thereby supporting better mental and physical well-being. This study highlights the importance of tackling ageing stereotypes to promote healthier ageing.

## Background

Ageism is the systematic stereotyping and discrimination of individuals based on their chronological age (Butler, 1969). As with other forms of discrimination and stereotyping, ageism can be internalised and, therefore, self-directed (Kornadt and Rothermund, 2012). Both when directed towards others or oneself, ageism springs from widespread stereotypes and marginalisation of older adults, with important repercussions on their quality of life and overall well-being (Kornadt et al., 2021). These ageist beliefs often lead to the homogenous characterisation of older adults, perpetuating misconceptions about their fragility, autonomy, and overall contribution to society (Walker, 2012). As a result, common but manageable medical conditions in later life, like chronic pain, depression, and sleep disorders, are often left untreated (Sarkisian et al., 2002). This linkage between negative images of ageing and a fatalistic view of older age as the only potential cause of illness in later life (Macé and Le Lec, 2011) further modulates older adults’ health-seeking behaviours (Levy and Apriceno, 2019). Such fatalistic approaches to health in older age negatively impact the probability of older adults engaging in preventive health practices and seeking medical treatment (Levy and Myers, 2004).

Sleep in older age is a complex multifactorial phenomenon in which normative age-related physiological changes (Li et al., 2018) are further modulated by shifts in routine due to retirement (Jing et al., 2020), individual circumstances linked to caregiving responsibilities (Kotronoulas et al., 2013), decreased exposure to natural light linked to changes in physical activity levels (Neikrug and Ancoli-Israel, 2010), and incidence of mental health conditions such as depression and anxiety (Gooneratne and Vitiello, 2014). Chronic sleep disruption across all age groups can lead to cognitive impairment (Bonnet and Arand, 2003; Teräs et al., 2023), with an increase in morbidity and mortality incidence among older adults (Khan-Hudson and Alessi, 2008), in addition to an overall increased risk of falls (Min and Slattum, 2018).

Despite extensive research on the impact of self-ageism on various dimensions of functioning (Hu et al., 2021), there are relatively few studies focusing on the role of negative age stereotypes in older adults’ health-seeking behaviours, particularly in response to chronic sleep disruption. Among the limited literature exploring this association, Sabatini et al.’s (2022) cross-sectional and longitudinal investigation identified significant associations between subjective sleep difficulties and self-perceptions of ageing. Similarly, Stephan et al. (2017) examined the relationship between subjective age and sleep in middle- and older-aged adults, revealing that those who felt younger than their chronological age reported better sleep quality. Yoon et al. (2023) also identified a connection between older subjective age and poor sleep quality in a population-based study, emphasising the impact of self-perceived ageing on sleep health. Building upon this body of work, Lin (2016) highlighted gender differences in self-perceptions of ageing and sleep among older adult Chinese residents in Taiwan, suggesting that cultural and demographic factors also play a role in this dynamic. These studies provide a foundational understanding of the complex interplay between ageing perceptions and sleep, underscoring the need for further investigation.

Widespread dysfunctional beliefs regarding the role of ageing are particularly present within the context of sleep disturbances (Robbins et al., 2019). Evidence suggests that older adults with poor sleep quality may hesitate to report such complaints due to the general belief that sleep difficulties are a normal part of ageing (Leblanc and Desjardins, 2015). This misconception also downplays the significance of sleep quality as a critical health concern (Morin, 2004). Although there are natural changes in sleep architecture in older age (i.e., reduced total sleep time and efficiency, increased sleep latency and duration of awakenings during the night; Miles and Dement, 1980), unrecognised pathologic patterns can represent a severe risk for mental well-being and quality of life of the person due to high comorbidity with physical and mental health conditions (Crowley, 2011).

These ageist beliefs can also foster specific dysfunctional sleep-related perceptions. For example, negative self-perceptions of ageing can trigger cognitive processes like catastrophising, where minor sleep difficulties are interpreted as severe or irreversible, reinforcing the belief that poor sleep is an inevitable part of ageing (Morin, 2004). Similarly, health engagement factors may influence sleep-related behaviours. Vincent and colleagues (Vincent et al., 2004) developed a questionnaire to evaluate how individuals attribute sleep-related experiences to external or internal factors. Known as Sleep Control beliefs, or Sleep Locus of Control (SLOC), these beliefs reflect whether individuals perceive their sleep patterns as driven by external forces or their own actions. Control beliefs usually decline with age, with an increased perception of control by external sources and a decreased personal sense of control and mastery (Lachman and Weaver, 1998).

Finally, emotional processes, such as anxiety or fear about ageing, can exacerbate sleep disturbances. Heightened physiological arousal stemming from these emotions may reinforce maladaptive sleep beliefs, further perpetuating a cycle of poor sleep and diminished well-being worsening sleep disturbances and fostering maladaptive sleep beliefs (Crowley, 2011; Gomez and Madey, 2001).

Considering the current but small evidence base on the detrimental effects of negative attitudes towards ageing on older adults’ well-being and the importance of sleep quality in later life (Ohayon, 2011), the aim of this paper is to examine potential associations between self-ageism in adults aged 60+ and their attitudes and beliefs towards sleep. Previous studies have emphasised the influence of self-directed age stereotypes on older adults’ health-seeking behaviours and overall health engagement (Smith et al., 2023). In this paper, we aim to explore this potential association within the context of the sleep domain by examining the synergies between beliefs about sleep and self-ageism in adults aged 60+.

## Study aims and objectives

This paper investigates the association between attitudes towards ageing and beliefs about sleep among adults aged 60+. It further explores the relationships between attitudes towards ageing, sleep locus of control, and dysfunctional beliefs about sleep. These dimensions are theoretically connected to older adults’ perceptions of ageing and influence subjective health evaluations.

It is hypothesised that:

1. Older adults with greater negative self-perceptions of ageing will report lower levels of internal sleep locus of control and higher levels of dysfunctional beliefs about sleep.
2. Older adults with greater negative self-perceptions of ageing tend to attribute sleep disruption to age decline and report higher levels of external sleep locus of control.
3. Older adults with greater negative self-perceptions of ageing tend to report higher levels of dysfunctional beliefs and attitudes towards sleep compared to those with positive self-perceptions.

## Methods

### Design

An online survey design was implemented to collect data. Predictor variables included measures of attitudes towards ageing. Outcome variables included self-reported measures of sleep quality, measures of dysfunctional beliefs towards sleep, and measures of sleep locus of control. Covariates included demographic characteristics, and measures of depression. Power analyses were conducted before data collection to determine a desirable sample size based on the requirements of the planned statistical tests. Power analyses were conducted by implementing GPower software version 3.1 (Wilson and Chaddha, 2009) to determine the minimum required sample size for the planned statistical analysis. Based on a power level of 0.95 power at a significance criterion of α = 0.05, the analysis indicated a minimum sample size of 138 participants to detect significant effects. Ethics Approval was obtained prior to the commencement of this study.

### Participants

A sample of 500 adults aged 60 years and above participated in this study. Inclusion criteria involved being aged 60 years and over and being fluent in English. The decision to use 60 years as the cut-off point for the minimum chronological age required derives from considerations regarding age-related changes in sleep among healthy individuals, as shown in various reviews and metanalysis (Ohayon et al., 2004). Participants were recruited through the online participant recruitment platform Prolific.^1^ Prolific enables large sample collection in compliance with social distancing and the General Data Protection Regulation (GDPR).

### Materials

#### Predictor variables

##### Attitudes towards ageing

Attitudes towards ageing were assessed through the brief version of Barker’s Ageing Perception Questionnaire (B-APQ; Sexton et al., 2014). This is a 5-point Likert scale containing 17 items, organised in the following five ageing perceptions domains: Timeline-Chronic (TC); Consequences Positive (PS); Control Positive (PC); Consequences and Control Negative (NC); and Emotional Representations (ER). For each domain, participants are asked to rate their level of agreement with different statements related to their personal experience of ageing. Example items for the subscales include Timeline-Chronic (TC), ‘I am always aware of the fact that I am getting older’; Consequences Positive (PS), ‘As I get older I get wiser’; Control Positive (PC) “The quality of my social life in later years depends on me”; Negative Control (NC) “I have no control over the effects which getting older has on my social life”; Emotional Representations (ER), “I feel angry when I think about getting older.”

Items in the scale Consequences and Control Negative are reverse scored. Overall, positive perceptions of ageing are indicated by higher scores in the PS, PC and NC subscales and low scores in the TC and ER subscales (Sexton et al., 2014). In this study, Alpha Cronbach for B-APQ global score is 0.677; its subscales are 0.723, 0.686, 0.803, 0.710 and 0.731. It is worth pointing out that the B-APQ global score and its subscale PS’s Cronbach Alpha < 0.7 (respectively 0.677 and 0.686), which is consistent with previous studies investigating similar age groups (Sexton et al., 2014; Brown et al., 2021; Jaafar et al., 2020).

### Outcome variables

#### Self-reported sleep quality

Pittsburgh Sleep Quality Index (PSQI; Buysse et al., 1989) is a self-reported questionnaire composed of 19 items which measure sleep quality and disturbances over 1 month preceding the survey. Example items include: “During the past month, how often have you had trouble sleeping because you wake up in the middle of the night or early morning?” and “During the past month, how would you rate your overall sleep quality?.” A final score greater than five is indicative of poor sleep quality. This instrument has proven to have high internal and global consistency while showing consistency across time and discriminating well between experimental/treatment and control groups (Buysse et al., 1989). In addition, this questionnaire is a valid psychometric measure to assess sleep quality across various populations (Andrykowski et al., 1997). Alpha Cronbach’s PSQI overall score is 0.735.

#### Dysfunctional beliefs towards sleep

The shortened version of the Dysfunctional Beliefs and Attitudes about Sleep Scale (DBAS-16; Vincent et al., 2004) was implemented to assess the participants’ dysfunctional beliefs and attitudes about sleep. Example items include: “I need 8 h of sleep to feel refreshed and function well during the day” and “When I sleep poorly on one night, I know it will disturb my sleep schedule for the whole week.’ Participants were asked to report their level of agreement with 16 statements that addressed widespread beliefs and attitudes related to sleep. This instrument implements a 10-point Likert scale ranging from 0 = Strongly disagree to 10 = Strongly Agree. The final global score was obtained by averaging the total scores of all items (i.e., adding scores for all 16 items and dividing by 16 for an average total score). Higher scores indicate a higher presence of dysfunctional beliefs and attitudes about sleep in the participants. In this study, Cronbach’s Alpha is 0.866.

#### Sleep locus of control

The Sleep Locus of Control scale (Vincent et al., 2004) is an 8-item questionnaire designed to assess how individuals attribute sleep-related experiences to factors outside their control or internal to their actions. A higher score on the SLOC indicates a greater internal attribution of sleep-related experiences, meaning that the individual believes their sleep quality is largely determined by their actions, behaviours, and personal control rather than external circumstances. Example items include: “I am directly responsible for my sleep” (internal locus of control) and “Good sleep is largely a matter of luck” (external locus of control). This uses a Likert scale ranging from 1 to 6, in which the respondents self-report their degree of agreement with different statements about their perception of control over their sleep. This scale has been shown to possess convergent validity with the internal subscale of the Multi-Dimensional Health Locus of Control Scale (MHLOC; Wallston et al., 1994) and good internal consistency (Vincent et al., 2004). Alpha Cronbach’s SLOC overall score is 0.781.

### Covariates

#### Demographic characteristics

Demographic data collected in this study were represented by age, gender, employability status, education level, subjective health, and presence of chronic medical conditions, all self-reported. Age was treated as a continuous variable, with the minimum allowed age by the inclusion criteria equal to 60 years of age. Gender was coded as 1 = Male, 2 = Female, 3 = Non-Binary/Third Gender. In our final sample, only one participant self-identified as ‘non-binary/third gender.’ To ensure meaningful and reliable results, we excluded this participant from the gender-based analysis due to the small sample size, which prevents meaningful group comparisons.

Employment Status was coded as 1 = Working (paid employee), 2 = Working (self-employed), 3 = Not working (temporary layoff from a job), 4 = Not working (looking for work), 5 = Not working (retired), 6 = Not working (disabled). Education was coded as 1 = Master or above, 2 = Bachelor’s degree, 3 = College, 4 = Trade school, and 5 = Secondary education.

#### Depression level

The presence of depressive symptomatology was assessed through the CES-D scale (Radloff, 1977). This scale has been used in several investigations focused on sleep in ageing (Reid et al., 2010; Rowe et al., 2008; Van Den Berg et al., 2008). The CES-D is a 20-item self-reported scale designed to assess depressive symptomatology in the general population. Example items include: “I was bothered by things that usually do not bother me “and “I felt hopeful about the future.” Global score ranges from 0 to 60, with higher scores indicating higher levels of depressive symptomatology. A global score equal to or greater than 16 is usually indicative of clinically significant depression (McDowell and Newell, 1996). Items 4, 8, 12 and 16 were reversed scored. In this study, Cronbach’s Alpha is 0.640. Although the results of CES-D Cronbach Alpha in this study are acceptable, even though moderate (Ursachi et al., 2015). Nevertheless, results involving this measure will be interpreted with caution, and further considerations will be included in the conclusion section of this paper.

### Procedure

Participants were asked to complete an online survey through the completion of Likert-type questions. Attention check questions were incorporated throughout the questionnaire, and the consistency of responses was examined. Participants first read an information sheet and completed a consent form. They then completed measures assessing attitudes towards ageing, sleep quality, dysfunctional sleep beliefs, sleep locus of control and depression. Following completion, they read a debrief form detailing the purpose of the study and their participation was concluded.

### Analysis

Data analysis was conducted using SPSS version 27. Reliability analyses were run to assess the internal consistency of the scales implemented in this study using Cronbach’s Alpha (α) while identifying a significance level of *p* < 0.05. Descriptive statistics were used to determine the frequency, mean, and standard deviations (SD) of the variables of interest. Pearson’s coefficient was used for interval variables, and hierarchical regression analysis was used for predicting criterion variables while controlling for the independent contributions of each variable of interest.

## Results

### Descriptive analysis

Participant distribution was male–female (50% vs. 49%) and employed-retired (39% vs. 54%), with 47% receiving bachelor’s or higher degrees. A more detailed breakdown is shown in Table 1. The sample reflects a WEIRD sample (i.e., White, Educated, Industrialised, Rich, and Democratic) (Casler et al., 2013), as most participants were highly educated and likely had good computer literacy due to the nature of the online recruitment platform used.

**Table 1:** Distribution of demographic data.

Variable	Frequency	Percentage
Gender		
Men	252	50.4%
Women	247	49.4%
Non-binary	1	0.2%
Employment status		
Working (paid employee)	141	28.2%
Working (self-employed)	54	10.8%
Not working (looking for work)	13	2.6%
Retired	268	53.6%
Not working (disabled)	8	1.6%
Not working (other)	16	3.2%
Educational level		
Masters’ degree or above	71	14.2%
Bachelor’s degree	167	33.4%
College	91	18.2%
Trade school	25	5%
Secondary education	142	28.4%
Other	4	0.8%

Table 2 summarises the descriptive statistics for the study variables, including chronological age, PSQI, SLOC, DBAS-16, B-APQ, and CES-D. The mean global PSQI score (7.12) suggests that participants, on average, experience sleep disturbances, as scores above 5 indicate poor sleep quality (Buysse et al., 1989). Other measures, such as SLOC (27.79) and DBAS-16 (3.94), reflect moderate perceptions of sleep control and dysfunctional beliefs about sleep, respectively, with no evidence of significant floor or ceiling effects across the scales. The CES-D mean score (11.17) indicates mild depressive symptoms, falling below the clinical concern threshold of 16. These patterns suggest the scales effectively captured variability in the sample.

**Table 2:** Continuous data mean, SD (i.e., standard deviation), and range.

Variable	Min-Max	Mean	SD
Age (years)	60–90	66.60	5.11
PSQI (Global)	0–17	7.12	3.58
SLOC (Global)	8–46	27.79	6.33
DBAS-16 (Global)	0.31–8.81	3.94	1.52
B-APQ (Global)	32–75	54.38	6.44
Timeline Chronic (TC)	3–15	8.56	2.36
Consequences Positive (CP)	4–15	11.24	1.95
Emotional Representations (EM)	3–15	7.86	2.71
Control Negative (NC)	5–23	14.31	3.42
Control Positive (PC)	3–15	12.4	1.87
CES-D (Global)	0–51	11.17	10.47

### Correlation analysis

As shown in Tables 3 and 4, significant associations were observed between B-APQ subscales and DBAS-16 and between B-APQ subscales and SLOC, indicating relationships between ageing perceptions, dysfunctional beliefs about sleep and sleep locus of control. Most B-APQ showed stronger correlations with DBAS-16 (e.g., Emotional Representations: R = 0.497, *p* < 0.001, reflecting 24.7% shared variance) compared toSLOC (e.g., Emotional Representations: R = −0.170, *p* < 0.001, reflecting 2.9% shared variance). This suggests that perceptions of ageing are more closely tied to dysfunctional beliefs about sleep than to sleep locus of control.

**Table 3:** Pearson’s correlation test between B-APQ global score, subscales, and DBAS-16.

B-APQ	DBAS-16				
	R	p-value	R^2^	95% Confidence Intervals (Lower – Upper)	
Global score	0.418	<0.001	0.175	0.343	0.488
Timeline Chronic (TC)	0.267	<0.001	0.071	0.184	0.347
Consequences Positive (PS)	−0.032	>0.05	0.001	−0.120	0.056
Emotional Representations (ER)	0.497	<0.001	0.247	0.428	0.561
Control Negative (NC)	0.330	<0.001	0.109	0.249	0.406
Control Positive (PC)	−0.207	<0.001	0.042	−0.289	−0.121

**Table 4:** Pearson’s correlations test between B-APQ global score, subscales, and SLOC.

B-APQ	SLOC				
	R	*p*-value	R^2^	95% Confidence Intervals (Lower – Upper)	
Global score	−0.106	<0.05	0.011	−0.192	−0.019
Timeline Chronic (TC)	−0.118	<0.05	0.014	−0.203	−0.030
Consequences Positive (PS)	0.130	<0.05	0.017	0.043	0.215
Emotional Representations (ER)	−0.170	<0.001	0.029	−0.254	−0.084
Control Negative (NC)	−0.198	<0.001	0.039	−0.281	−0.113
Control Positive (PC)	0.222	<0.001	0.049	0.137	0.304

In terms of direction, positive ageing perceptions, such as Control Positive (PC), were negatively associated with DBAS-16 (R = −0.207, *p* < 0.001) and positively associated with SLOC (R = 0.222, *p* < 0.001), indicating that individuals with more positive ageing beliefs tend to have fewer dysfunctional sleep beliefs and a stronger sense of control over sleep. Conversely, negative perceptions, such as Emotional Representations (ER) and Control Negative (NC), were positively associated with DBAS-16 and negatively associated with SLOC, indicating that negative ageing beliefs align with more dysfunctional sleep beliefs and less perceived control over sleep. These correlations were further explored via hierarchical regression analyses, discussed next.

### Regression analysis

Hierarchical regression analyses were implemented to investigate further a potential relationship between self-perceptions of ageing and, respectively, dysfunctional beliefs towards sleep and sleep locus of control. Assumptions for this test were checked using a scatterplot of standardised residuals and standardised predicted values, the Durbin-Watson test for independence of observation, and the normality of residuals using a histogram of residuals. Cook’s test was conducted to verify the presence of outliers. Multicollinearity was checked by examining tolerance and the variance inflation factor (VIF).

In the first regression analysis, Model 1 includes DBAS-16 as a control variable for its potential influence on sleep locus of control (SLOC). This decision was theoretically driven, based on literature suggesting that dysfunctional beliefs about sleep can affect perceived control over sleep (Morin et al., 2007). In contrast, SLOC was not included in Supplementary Table 1 as a control variable because the primary focus of that analysis was to examine the impact of self-perceptions of ageing on dysfunctional beliefs about sleep, not on the sleep locus of control itself. Thus, SLOC was not considered a necessary control variable for that analysis.

First, to verify how much of DBAS-16’s variance can be accounted for by B-APQ’s subscales, hierarchical regression analysis with CES-D, PSQI, and demographic variables (i.e., age, gender, employment status, and education level) as control variables was conducted (see Supplementary Table 1 for a detailed report of the analysis). Dummy coding included nominal variables such as employment status and education level in the regression models, ensuring that the analysis appropriately handled the categorical nature of these predictors.

The first model included age, gender, employment status, education level, CES-D, and PSQI global score as control variables. This model explained 29.5% of the variance in DBAS-16, R^2^ = 0.295, Adjusted R^2^ = 0.275, *F*(14, 484) = 14.497, *p* < 0.001. The second model included the control variables plus Emotional Representations (ER). This model explained 35.9% of the variance in DBAS-16, R^2^ = 0.359, Adjusted R^2^ = 0.339, *F*(15, 483) = 18.065, *p* < 0.001. The addition of ER significantly increased explained variance, ΔR^2^ = 0.064, *F*(1, 483) = 48.212, *p* < 0.001. The rationale for entering Emotional Representations (ER) in the second model was based on theoretical considerations. Prior literature suggests that emotional responses to ageing, such as anxiety or fear, primarily influence health-related beliefs and behaviours (Levy and Myers, 2004; Gomez and Madey, 2001). Thus, ER was entered first to examine its unique contribution to dysfunctional beliefs about sleep. The remaining B-APQ subscales (excluding Positive Consequences) were added in the third model to determine whether they accounted for additional variance over and above the effect of ER.

Finally, the third model included the control variables, ER, and the remaining B-APQ subscales. This model explained 38.4% of the variance in DBAS-16, R^2^ = 0.384, Adjusted R^2^ = 0.359, *F*(19, 479) = 15.683, *p* < 0.001. Adding the B-APQ subscales significantly increased explained variance, ΔR^2^ = 0.024, *F*(4, 479) = 4.686, *p* = 0.001. Notably, the Positive Consequences (PS) subscale showed a significant effect in the regression analysis (*p* < 0.05) despite its non-significant negative correlation with DBAS-16 in the Pearson correlation. This suggests a possible suppression or interaction effect that may warrant further exploration.

A similar regression was conducted to examine how much of the variance in SLOC can be explained by B-APQ’s subscales while controlling for demographic variables (age, gender, employment status, and education level), PSQI global score, CES-D, and DBAS-16 (see Supplementary Table 2 for a detailed report of the analysis).

The first model included age, gender, employment status, education level, PSQI global score, CES-D, and DBAS-16 as control variables. This model explained 19.5% of the variance in SLOC, R^2^ = 0.195, Adjusted R^2^ = 0.170, *F*(15, 483) = 7.785, *p* < 0.001.

The second model included the control variables plus Negative Control (NC) and Positive Control (PC). This model explained 24.4% of the variance in SLOC, R^2^ = 0.244, Adjusted R^2^ = 0.218, *F*(17, 481) = 9.146, *p* < 0.001. Adding NC and PC significantly increased the explained variance, ΔR^2^ = 0.050, *F*(2, 481) = 15.779, *p* < 0.001. In Supplementary Table 2, Model 2 includes the Negative Control (NC) and Positive Control (PC) subscales, with the remaining subscales entered in Model 3. This order was chosen to explore the unique contribution of perceived control (both positive and negative) over ageing-related changes, which is considered a critical factor in shaping sleep locus of control (Lachman and Weaver, 1998). The remaining subscales were included in Model 3 to determine if they added any explanatory power beyond the control variables.

Finally, the third model included the control variables, NC, PC, and the remaining B-APQ subscales (TC, ER, and PS). This model explained 25.1% of the variance in SLOC, R^2^ = 0.251, Adjusted R^2^ = 0.220, *F*(20, 478) = 8.027, *p* < 0.001. Adding the remaining B-APQ subscales did not result in a significant increase in explained variance, ΔR^2^ = 0.007, *F*(3, 478) = 1.519, *p* = 0.209.

## Discussion

This study investigated the associations between self-perceptions of ageing and sleep-related attitudes and beliefs among adults aged 60+ years, focusing on sleep locus of control (SLOC) and dysfunctional beliefs about sleep (DBAS-16). Consistent with our study’s hypotheses, negative self-perceptions of ageing, particularly emotional responses to ageing, were associated with higher levels of dysfunctional beliefs about sleep. Positive control beliefs about ageing were positively associated with an internal sleep locus of control, while negative control beliefs were associated with an external sleep locus of control. These findings suggest that attitudes towards ageing play a significant role in shaping sleep-related beliefs and perceptions in older adults.

Key findings from this study align with and extend existing research on self-perception of ageing and health outcomes. For example, previous studies have highlighted how negative emotional responses to ageing, such as anxiety or fear, are linked to maladaptive coping strategies and poorer health outcomes (Gomez and Madey, 2001). Similarly, in this study, older adults reporting negative emotional responses to ageing demonstrated higher levels of dysfunctional beliefs about sleep, which are known to exacerbate sleep difficulties (Morin et al., 2007). These results reinforce the importance of addressing emotional responses to ageing in promoting healthier sleep attitudes and behaviours.

In a broader context of the literature, these findings emphasise the critical role of control beliefs in sleep health. Consistent with past research showing that an internal locus of control is associated with better sleep quality and health outcomes (Stephan et al., 2018), this study highlights how positive control beliefs about ageing may bolster a sense of agency over sleep. Interventions aimed at fostering positive self-perceptions of ageing could enhance older adults’ sense of control over their sleep, improving sleep management and encouraging proactive health-seeking behaviours. Conversely, addressing negative control beliefs may help mitigate feelings of helplessness and external dependence, which can contribute to poorer sleep outcomes (Vincent et al., 2004).

From a practical perspective, these findings underscore the potential for targeted interventions to promote positive attitudes toward ageing and challenge age-related stereotypes. By incorporating strategies that enhance emotional resilience and reinforce positive control beliefs, health programmes could address both ageing-related concerns and sleep disturbances, ultimately supporting healthier ageing.

In conclusion, this study adds to the growing body of evidence highlighting the interplay between self-perception of ageing and sleep health. By identifying specific attitudes and beliefs that influence sleep in older adults, these findings provide a foundation for developing targeted interventions to improve both sleep and overall well-being in the ageing population.

## Limitations and future directions

This study’s results need to be considered in light of its potential limitations. Firstly, the sample’s age (i.e., Mean 66.6 years) is relatively young, and online recruitment of the participants might have affected the generalisability of the results. Future studies could consider targeted sampling of older age groups to address this.

Longitudinal studies are needed to explore these potential bidirectional relationships further, allowing for a more robust understanding of how changes in attitudes towards ageing might affect sleep beliefs and behaviours over time. Additionally, future research should investigate the underlying mechanisms that could explain the associations observed in this study. For example, negative self-perceptions of ageing may lead to greater stress or anxiety about sleep, which in turn could contribute to more dysfunctional sleep beliefs. Alternatively, positive perceptions of control over ageing might promote adaptive coping strategies that enhance an internal locus of control over sleep. A longitudinal design would allow for the examination of these potential mediating pathways, helping to clarify how psychological factors related to ageing contribute to sleep health among older adults.

The CES-D scale used in this study demonstrated a Cronbach’s alpha of 0.640, which, although acceptable, indicates moderate reliability. This lower reliability suggests that the measure may not have fully captured the range of depressive symptoms in our sample, potentially affecting the strength of associations with other variables. Future research should consider using alternative or supplementary measures to assess depressive symptoms more comprehensively. This scale has been implemented as a control variable in the hierarchical regression analyses conducted between B-APQ and, respectively, SLOC and DBAS-16, its moderately low Cronbach Alpha (i.e., 0.640) might have affected this set of analyses. Nevertheless, we decided to keep this scale in our analyses since, as discussed above, CES-D’s Alpha, even though moderate, is acceptable (Ursachi et al., 2015).

Additionally, participants were not controlled for previously diagnosed sleep disorders nor their treatment (e.g., through CBT-I, Cognitive Behavioural Therapy for Insomnia), nor was information collected regarding the use of medication for sleep, which can be a significant external factor influencing sleep beliefs and behaviours. The absence of this information limits our ability to distinguish between internal versus external sources of perceived control over sleep (Vincent et al., 2004). Other relevant measures such as participants’ physical activity rate, patterns of morningness and eveningness, and daytime napping, all measures shown to affect sleep quality in ageing (Ancoli-Israel and Martin, 2006; Kim et al., 2010; Merikanto and Partonen, 2020; Vanderlinden et al., 2020), can also be collected and analysed in future studies to extend our results.

Sociodemographic measures such as ethnicity and marital status were not recorded. Furthermore, we did not assess whether participants were recently separated from a spouse due to death, divorce, or other circumstances. Living arrangements, such as living alone, with family (either joint or nuclear), or in community housing for older adults, were likewise not documented, despite their potential influence on psychological well-being and sleep (Chu et al., 2022). Finally, the study did not consider physical, mental, or cognitive disabilities (e.g., locomotor limitations, memory impairments, or reduced attention) that may emerge with age and influence both perceptions of ageing and sleep-related experiences (Okoye et al., 2021).

These measures might offer a deeper insight into factors shaping attitudes towards ageing and sleep, and future work to incorporate such variables may yield additional dynamics.

## Conclusion

This study provides new insights into how attitudes toward ageing are linked to sleep-related beliefs and behaviours in later life. Key findings demonstrate that negative emotional responses to ageing are associated with maladaptive beliefs about sleep, while positive control beliefs enhance an internal locus of control over sleep. These findings suggest potential targets for interventions that address both emotional and control dimensions of ageing attitudes.

Addressing age-related stereotypes and fostering positive perceptions of ageing could promote healthier sleep behaviours and improve overall well-being in older adults. Future research should explore these associations further, focusing on diverse populations and employing longitudinal methods to clarify causal pathways and inform policy and practice.

## Data Availability

The raw data supporting the conclusions of this article will be made available by the authors, without undue reservation.

## Footnotes

^1^ www.prolific.co
