# Supplementary material for: Retiring for the night: how negative attitudes towards ageing can shape expectations and attitudes towards sleep among adults aged 60+
Source: Front Psychol. 2025 Apr 30;16:1556248. doi: 10.3389/fpsyg.2025.1556248 (PMC12075417; doi:10.3389/fpsyg.2025.1556248)
Supplement: Supplementary file 1 [file Supplementary_file_1.docx]

**Appendix 1:** Hierarchical Regression Models with PSQI, CESD and Chronological Age as control (Model 1), ER (Model 2) and ER, TC, NC and PC (Model 3) of DBAS-16.

| DBAS-16  (Dependent variable) | | MODEL 1 | | | | | Collinearity Statistics | |
| --- | --- | --- | --- | --- | --- | --- | --- | --- |
|  | | B | SE B | C.I. | t | *p* | Tolerance | VIF |
| (Constant) | | 4.592 | .859 | [2.903, 6.280] | 5.343 | <.001 |  |  |
| Age | | -.031 | .013 | [-.057, -.005] | -2.351 | <.05 | .744 | 1.345 |
| Gender (Female) | | .045 | .120 | [-.191, -.281] | .375 | >.05 | .928 | 1.078 |
| Employment status (Self-employed) | | .155 | .211 | [-.259, .570] | .736 | >.05 | .779 | 1.283 |
| Employment status (Looking for work) | | .067 | .381 | [-.681, .815] | .176 | >.05 | .910 | 1.099 |
| Employment status (Retired) | | -.073 | .152 | [-.371, .225] | -.482 | >.05 | .586 | 1.707 |
| Employment status (Disabled) | | .108 | .476 | [-.827, 1.044] | .227 | >.05 | .935 | 1.070 |
| Employment status (Prefer not to say) | | -.047 | .347 | [-.729, .635] | -.135 | >.05 | .895 | 1.118 |
| Education level (Bachelor’s degree) | | .052 | .185 | [-.312, .417] | .283 | >.05 | .438 | 2.283 |
| Education level (College) | | .037 | .208 | [-.372, .446] | .179 | >.05 | .517 | 1.933 |
| Education level (Trade school) | | -.002 | .306 | [-.604, .599] | -.008 | >.05 | .749 | 1.335 |
| Education level (Secondary education) | | .175 | .191 | [-.201, -.551] | .912 | >.05 | .449 | 2.229 |
| Education level (Other) | | .951 | .667 | [-.360, 2.262] | 1.425 | >.05 | .945 | 1.059 |
| CESD | | .047 | .007 | [.034, .060] | 7.237 | <.001 | .723 | 1.384 |
| PSQI global | | .113 | .019 | [.076, .150] | 6.022 | <.001 | .734 | 1.361 |
|  |  |  | | | | | | |
| Model Fit | | F (14, 484) = 14,497, *p* < .001 | | | | | | |
| Adjusted R^2^ | | .275 | | | | | | |
|  |  |  |  |  |  |  |  |  |
|  |  | MODEL 2 | | | | | Collinearity Statistics | |
|  |  | B | SE B | C.I. | t | *p* | Tolerance | VIF |
| (Constant) | | 3.098 | .848 | [1.432, 4.765] | 3.654 | <.001 |  |  |
| Age | | -.023 | .013 | [-.048, .002] | -1.834 | >.05 | .738 | 1.356 |
| Gender (Female) | | .000 | .115 | [-.225, .226] | .003 | >.05 | .925 | 1.081 |
| Employment status (Self-employed) | | .137 | .201 | [-.259, .532] | .679 | >.05 | .779 | 1.283 |
| Employment status (Looking for work) | | .052 | .363 | [-.662, .765] | .142 | >.05 | .910 | 1.099 |
| Employment status (Retired) | | -.116 | .145 | [-.400, .169] | -.800 | >.05 | .585 | 1.710 |
| Employment status (Disabled) | | .162 | .455 | [-.731, 1.056] | .357 | >.05 | .935 | 1.070 |
| Employment status (Prefer not to say) | | -.156 | .332 | [-.808, .495] | -.471 | >.05 | .893 | 1.120 |
| Education level (Bachelor’s degree) | | .035 | .177 | [-.313, .383] | .197 | >.05 | .438 | 2.284 |
| Education level (College) | | .054 | .199 | [-.336, .445] | .274 | >.05 | .517 | 1.933 |
| Education level (Trade school) | | .001 | .292 | [-.574, .575] | .002 | >.05 | .749 | 1.335 |
| Education level (Secondary education) | | .127 | .183 | [-.232, .486] | .694 | >.05 | .448 | 2.232 |
| Education level (Other) | | .790 | .637 | [-.463, 2.042] | 1.239 | >.05 | .943 | 1.060 |
| CESD | | .024 | .007 | [.010, .037] | 3.323 | <.001 | .557 | 1.797 |
| PSQI global | | .104 | .018 | [.069, .140] | 5.793 | <.001 | .731 | 1.396 |
| E.R. (B- APQ subscale) | | .174 | .025 | [.125, .223] | 6.944 | <.001 | .662 | 1.510 |
|  |  |  |  |  |  |  |  |  |
| Model Fit | | F(15, 483) = 18.065, *p* < .001 | | | | | | |
| Adjusted R^2^ | | .339 | | | | | | |
| R^2^ Change | | .064 | | | | | | |
|  |  |  |  |  |  |  |  |  |
|  |  | MODEL 3 | | | | | Collinearity Statistics | |
|  |  | B | SE B | C.I. | t | *p* | Tolerance | VIF |
| (Constant) | | 1.800 | 1.038 | [-.239, 3.839] | 1.735 | >.05 |  |  |
| Age | | -.021 | .013 | [-.045, .004] | -1.658 | >.05 | .719 | 1.391 |
| Gender (Female) | | .024 | .116 | [-.203, .251] | .210 | >.05 | .887 | 1.128 |
| Employment status (Self-employed) | | .102 | .200 | [-.291, .494] | .509 | >.05 | .767 | 1.303 |
| Employment status (Looking for work) | | .067 | .359 | [-.638, .772] | .186 | >.05 | .906 | 1.104 |
| Employment status (Retired) | | -.115 | .144 | [-.397, .167] | -.800 | >.05 | .578 | 1.731 |
| Employment status (Disabled) | | .046 | .449 | [-.837, .929] | .102 | >.05 | .928 | 1.077 |
| Employment status (Prefer not to say) | | -.037 | .332 | [-.689, .616] | -.110 | >.05 | .865 | 1.156 |
| Education level (Bachelor’s degree) | | -.013 | .176 | [-.358, .332] | -.075 | >.05 | .431 | 2.318 |
| Education level (College) | | .045 | .196 | [-.341, .431] | .229 | >.05 | .515 | 1.943 |
| Education level (Trade school) | | -.026 | .288 | [-.592, .541] | -.089 | >.05 | .747 | 1.338 |
| Education level (Secondary education) | | .068 | .181 | [-.288, .424] | .374 | >.05 | .442 | 2.260 |
| Education level (Other) | | .637 | .632 | [-.606, 1.879] | 1.007 | >.05 | .930 | 1.075 |
| CESD | | .023 | .007 | [.009, .038] | 3.209 | .001 | .505 | 1.981 |
| PSQI global | | .103 | .018 | [.068, .138] | 5.795 | <.001 | .725 | 1.379 |
| E.R. (B-APQ subscale) | | .158 | .027 | [.106, .211] | 5.931 | <.001 | .566 | 1.766 |
| T.C. (B-APQ subscale) | | .005 | .029 | [-.051, .062] | .185 | >.05 | .645 | 1.550 |
| P.S. (B-APQ subscale) | | .104 | .030 | [.044, .163] | 3.404 | <.001 | .845 | 1.184 |
| N.C. (B-APQ subscale) | | .049 | .025 | [-.001, .098] | 1.923 | >.05 | .617 | 1.621 |
| P.C. (B-APQ subscale) | | -.055 | .033 | [-.119, .009] | -1.691 | >.05 | .800 | 1.249 |
|  |  |  |  |  |  |  |  |  |
| Model Fit | | F(19, 479) = 15.683, *p* < .001 | | | | | | |
| Adjusted R^2^ | | .359 | | | | | | |
| R^2^ Change | | .024 | | | | | | |

**Appendix 2:** Hierarchical Regression Models with PSQI, CESD and DBAS-16 as control variables (Model 1), NC and PC (Model 2) and NC, PC, TC, ER and PS (Model 3) of SLOC

| SLOC  (Dependent variable) | | MODEL 1 | | | | | Collinearity Statistics | |
| --- | --- | --- | --- | --- | --- | --- | --- | --- |
|  | | B | SE B | C.I. | t | *p* | Tolerance | VIF |
| (Constant) | | 30.520 | 3.945 | [22.769, 38.270] | 7.737 | <.001 |  |  |
| Age | | .039 | .059 | [-.077, .155] | .659 | >.05 | .735 | 1.360 |
| Gender (Female) | | -2.213 | .536 | [-3.266, -1.161] | -4.131 | <.001 | .928 | 1.078 |
| Employment status (Self-employed) | | .683 | .941 | [-1.167, 2.532] | .725 | >.05 | .778 | 1.285 |
| Employment status (Looking for work) | | .743 | 1.698 | [-2.593, 4.079] | .438 | >.05 | .910 | 1.099 |
| Employment status (Retired) | | -.482 | .676 | [-1.811, .847] | -.713 | >.05 | .586 | 1.708 |
| Employment status (Disabled) | | 1.334 | 2.124 | [-2.840, 5.509] | .628 | >.05 | .935 | 1.070 |
| Employment status (Prefer not to say) | | -1.907 | 1.548 | [-4.949, 1.135] | -1.232 | >.05 | .895 | 1.118 |
| Education level (Bachelor’s degree) | | 1.338 | .827 | [-.288, 2.964] | 1.617 | >.05 | .438 | 2.284 |
| Education level (College) | | 2.166 | .929 | [.341, 3.991] | 2.332 | <.05 | .517 | 1.933 |
| Education level (Trade school) | | .244 | 1.366 | [-2.441, 2.929] | .179 | >.05 | .749 | 1.335 |
| Education level (Secondary education) | | 1.754 | .854 | [.076, 3.433] | 2.054 | <.05 | .448 | 2.232 |
| Education level (Other) | | -2.069 | 2.983 | [-7.929, 3.792] | -.694 | >.05 | .941 | 1.063 |
| CESD | | -.001 | .031 | [-.061, .059] | -.040 | >.05 | .652 | 1.533 |
| PSQI global | | -.567 | .087 | [-.738, -.396] | -6.512 | <.001 | .683 | 1.463 |
| DBAS-16 | | -.322 | .203 | [-.720, .077] | -1.587 | >.05 | .705 | 1.419 |
|  |  |  | | | | | | |
| Model Fit | | F(15, 483) = 7.785, *p* < .001 | | | | | | |
| Adjusted R^2^ | | .170 | | | | | | |
|  |  |  |  |  |  |  |  |  |
|  |  | MODEL 2 | | | | | Collinearity Statistics | |
|  |  | B | SE B | C.I. | t | *p* | Tolerance | VIF |
| (Constant) | | 28.471 | 4.588 | [19.456, 37.486] | 6.206 | <.001 |  |  |
| Age | | .022 | .057 | [-.090, .135] | .389 | >.05 | .730 | 1.370 |
| Gender (Female) | | -2.625 | .525 | [-3.657, -1.592] | -4.995 | <.001 | .909 | 1.100 |
| Employment status (Self-employed) | | .748 | .914 | [-1.048, 2.545] | .819 | >.05 | .778 | 1.286 |
| Employment status (Looking for work) | | 1.019 | 1.649 | [-2.222, 4.259] | .618 | >.05 | .909 | 1.100 |
| Employment status (Retired) | | -.179 | .659 | [-1.474, 1.115] | -.272 | >.05 | .581 | 1.721 |
| Employment status (Disabled) | | 1.890 | 2.067 | [-2.171, 5.951] | .915 | >.05 | .931 | 1.074 |
| Employment status (Prefer not to say) | | -1.696 | 1.514 | [-4.672, 1.279] | -1.120 | >.05 | .881 | 1.135 |
| Education level (Bachelor’s degree) | | 1.322 | .804 | [-.259, 2.902] | 1.643 | >.05 | .437 | 2.290 |
| Education level (College) | | 2.067 | .903 | [.292, 3.842] | 2.289 | <.05 | .516 | 1.940 |
| Education level (Trade school) | | .256 | 1.327 | [-2.351, 2.864] | .193 | >.05 | .749 | 1.336 |
| Education level (Secondary education) | | 1.912 | .830 | [.281, 3.543] | 2.304 | <.05 | .447 | 2.236 |
| Education level (Other) | | -.869 | 2.903 | [-6.574, 4.835] | -.299 | >.05 | .936 | 1.069 |
| CESD | | .049 | .031 | [-.012, .110] | 1.575 | >.05 | .592 | 1.691 |
| PSQI global | | -.600 | .085 | [-.767, -.434] | -7.076 | <.001 | .678 | 1.474 |
| DBAS-16 | | -.121 | .201 | [-.516, .274] | -.600 | >.05 | .675 | 1.482 |
| N.C. (B-APQ subscale) | | -.310 | .106 | [-.519, -.102] | -2.925 | <.05 | .743 | 1.346 |
| P.C. (B-APQ subscale) | | .563 | .147 | [.273, .852] | 3.821 | <.001 | .830 | 1.204 |
|  |  |  |  |  |  |  |  |  |
| Model Fit | | F(2, 481) = 15.779, *p* < .001 | | | | | | |
| Adjusted R^2^ | | .218 | | | | | | |
| R^2^ Change | | .050 | | | | | | |
|  |  |  |  |  |  |  |  |  |
|  |  | MODEL 3 | | | | | Collinearity Statistics | |
|  |  | B | SE B | C.I. | t | *p* | Tolerance | VIF |
| (Constant) | | 25.685 | 4.786 | [16.281, 35.090] | 5.367 | <.001 |  |  |
| Age | | .032 | .058 | [-.082, .145] | .547 | >.05 | .715 | 1.399 |
| Gender (Female) | | -2.757 | .531 | [-3.801, -1.714] | -5.192 | <.001 | .887 | 1.128 |
| Employment status (Self-employed) | | .633 | .919 | [-1.173, 2.439] | .689 | >.05 | .767 | 1.304 |
| Employment status (Looking for work) | | 1.179 | 1.649 | [-2.062, 4.420] | .715 | >.05 | .906 | 1.104 |
| Employment status (Retired) | | -.182 | .660 | [-1.479, 1.115] | -.276 | >.05 | .577 | 1.734 |
| Employment status (Disabled) | | 1.926 | 2.066 | [-2.134, 5.985] | .932 | >.05 | .928 | 1.077 |
| Employment status (Prefer not to say) | | -1.654 | 1.526 | [-4.653, 1.345] | -1.084 | >.05 | .865 | 1.156 |
| Education level (Bachelor’s degree) | | 1.145 | .808 | [-.443, 2.732] | 1.417 | >.05 | .431 | 2.318 |
| Education level (College) | | 1.999 | .903 | [.225, 3.772] | 2.215 | <.05 | .515 | 1.943 |
| Education level (Trade school) | | .184 | 1.326 | [-2.421, 2.789] | .139 | >.05 | .747 | 1.338 |
| Education level (Secondary education) | | 1.798 | .833 | [.161, 3.435] | 2.158 | <.05 | .442 | 2.261 |
| Education level (Other) | | -.996 | 2.910 | [-6.715, 4.722] | -.342 | >.05 | .928 | 1.077 |
| CESD | | .050 | .034 | [-.017, .116] | 1.458 | >.05 | .494 | 2.023 |
| PSQI global | | -.605 | .085 | [-.771, -.438] | -7.140 | <.001 | .678 | 1.475 |
| DBAS-16 | | -.236 | .210 | [-.649, .177] | -1.123 | >.05 | .616 | 1.622 |
| N.C. (B-APQ subscale) | | -.332 | .117 | [-.561, -.103] | -2.849 | <.05 | .612 | 1.633 |
| P.C. (B-APQ subscale) | | .504 | .150 | [.209, .799] | 3.356 | <.001 | .796 | 1.257 |
| T.C. (B-APQ subscale) | | -.033 | .132 | [-.293, .226] | -.251 | >.05 | .645 | 1.550 |
| E.R. (B-APQ subscale) | | .166 | .127 | [-.084, .416] | 1.306 | >.05 | .527 | 1.896 |
| P.S. (B-APQ subscale) | | .257 | .142 | [-.021, .535] | 1.814 | >.05 | .825 | 1.213 |
|  |  |  |  |  |  |  |  |  |
| Model Fit | | F(20, 478) = 8.027, *p* < .001 | | | | | | |
| Adjusted R^2^ | | .220 | | | | | | |
| R^2^ Change | | .007 | | | | | | |
